# Benign Metastasizing Leiomyoma in a Patient With No Known History of Uterine Leiomyomas

**DOI:** 10.7759/cureus.68314

**Published:** 2024-08-31

**Authors:** Dhruva Kadiyala, Morgan Sly, Joseph Montecalvo, Dharshan Vummidi

**Affiliations:** 1 Department of Radiology, Wayne State University School of Medicine, Detroit, USA; 2 Department of Radiology, Henry Ford Health System, Detroit, USA; 3 Department of Pathology, Henry Ford Health System, Detroit, USA

**Keywords:** gynecology and oncology, radiology, extrauterine fibroids, thoracic imaging, pulmonary benign metastasizing leiomyoma

## Abstract

Benign metastasizing leiomyoma (BML) is a rare medical condition characterized by metastasis of fibroid tissue from uterine leiomyomas to other areas of the body, most commonly the lungs. While BML is mostly prevalent in women with a prior history of uterine leiomyomas who underwent surgical intervention, this case report explores the case of a 50-year-old female who was diagnosed with pulmonary benign metastasizing leiomyoma (PBML) with no prior history of confirmed leiomyomas. After initially presenting with worsening cough and congestion, chest radiograph and computed tomography revealed multiple bilateral pulmonary nodules, initially raising concerns for malignancy. Further, a workup with bronchoscopy with fine needle aspiration and pulmonary lesion biopsy revealed the presence of smooth muscle tissue suggestive of PBML. Subsequent uterine ultrasonography revealed a 3-cm intramural uterine fibroid, supporting the diagnosis. This case highlights the diagnostic challenge posed by PBML due to its asymptomatic manifestation and radiological similarity with other serious conditions such as malignancy and sarcoidosis. The case further highlights the importance of recognizing typical radiological features of PBML and the necessity of histological examination for accurate diagnosis. Finally, the critical role of a multidisciplinary approach in managing such rare conditions and the need for individualized treatment are also explored.

## Introduction

Benign metastasizing leiomyoma (BML) is a rare condition, with less than 150 cases documented in the literature as of 2020 [1]. BML typically affects women of reproductive age and is characterized by the presence of smooth muscle tissue that has metastasized from uterine fibroids to other areas of the body, most commonly the lungs [2]. BML is often asymptomatic, with lesions typically discovered incidentally in patients with a known history of leiomyomas who underwent myomectomy or hysterectomy. When symptoms do occur, they can include cough, chest pain, and shortness of breath [2]. As of 2017, there are only 10 documented cases of BML in women without prior leiomyoma surgery [3]. Of these 10, just three patients received a BML diagnosis with no known history of leiomyomas [3-6]. BML with metastatic pulmonary lesions, which can be referred to as pulmonary benign metastatic leiomyoma (PBML), is a condition that poses a significant diagnostic challenge as the pulmonary lesions often mimic the radiological findings of more common, sometimes grave, pulmonary conditions, including malignancy [2]. With such significant overlap in findings, it is imperative that radiologists recognize typical radiological features of PBML and recommend subsequent confirmatory testing to ensure a timely and accurate diagnosis. This report describes a patient who was diagnosed with PBML with no previous history of leiomyoma after careful evaluation for multiple pulmonary lesions discovered on a chest radiograph performed for worsening cough and congestion.

## Case presentation

**Figure 1:**
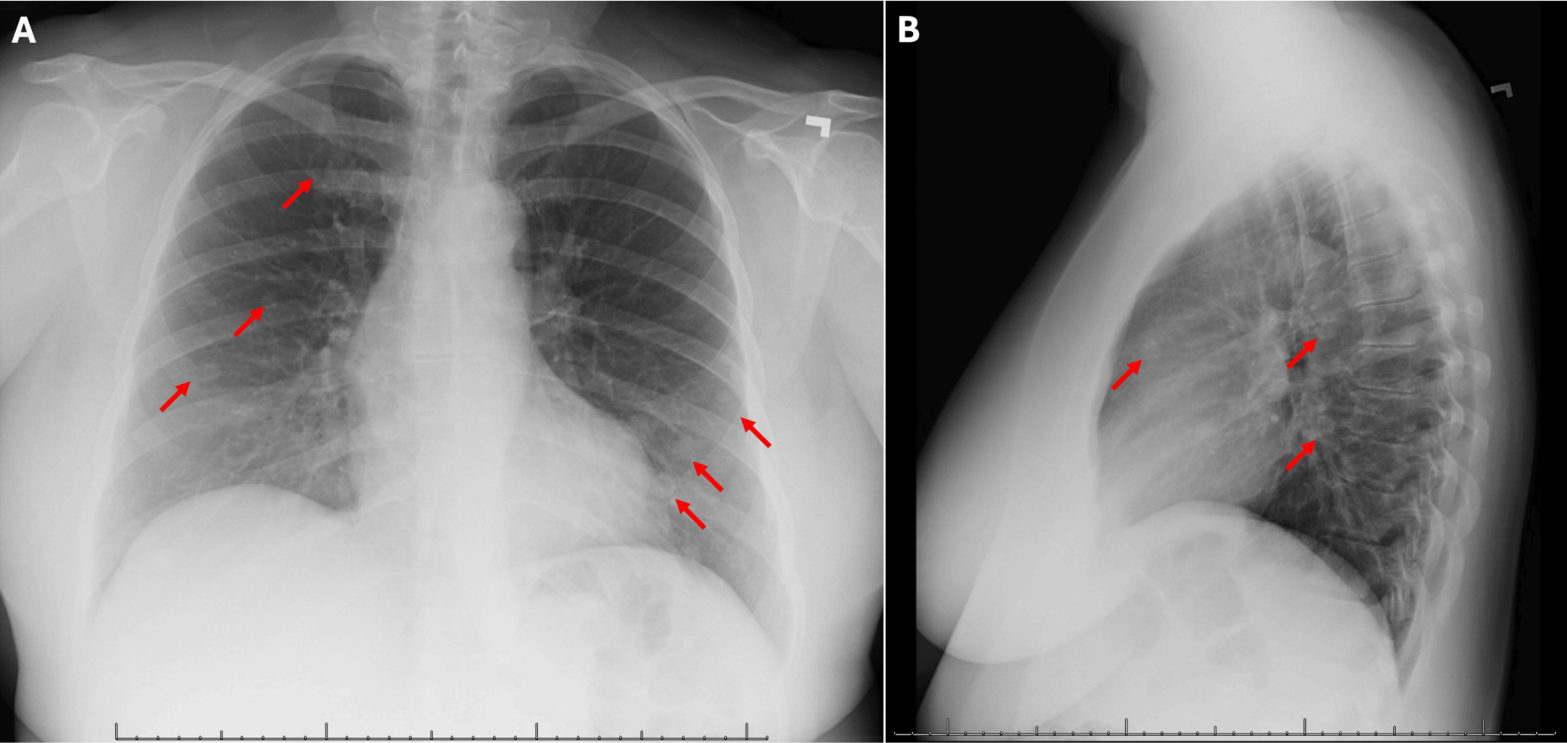
Chest X-ray imaging demonstrating diffuse bilateral pulmonary nodules. (a) Posterior anterior radiographic view. (b) Lateral radiographic view. Representative pulmonary nodules are indicated using red arrows.

**Figure 2:**
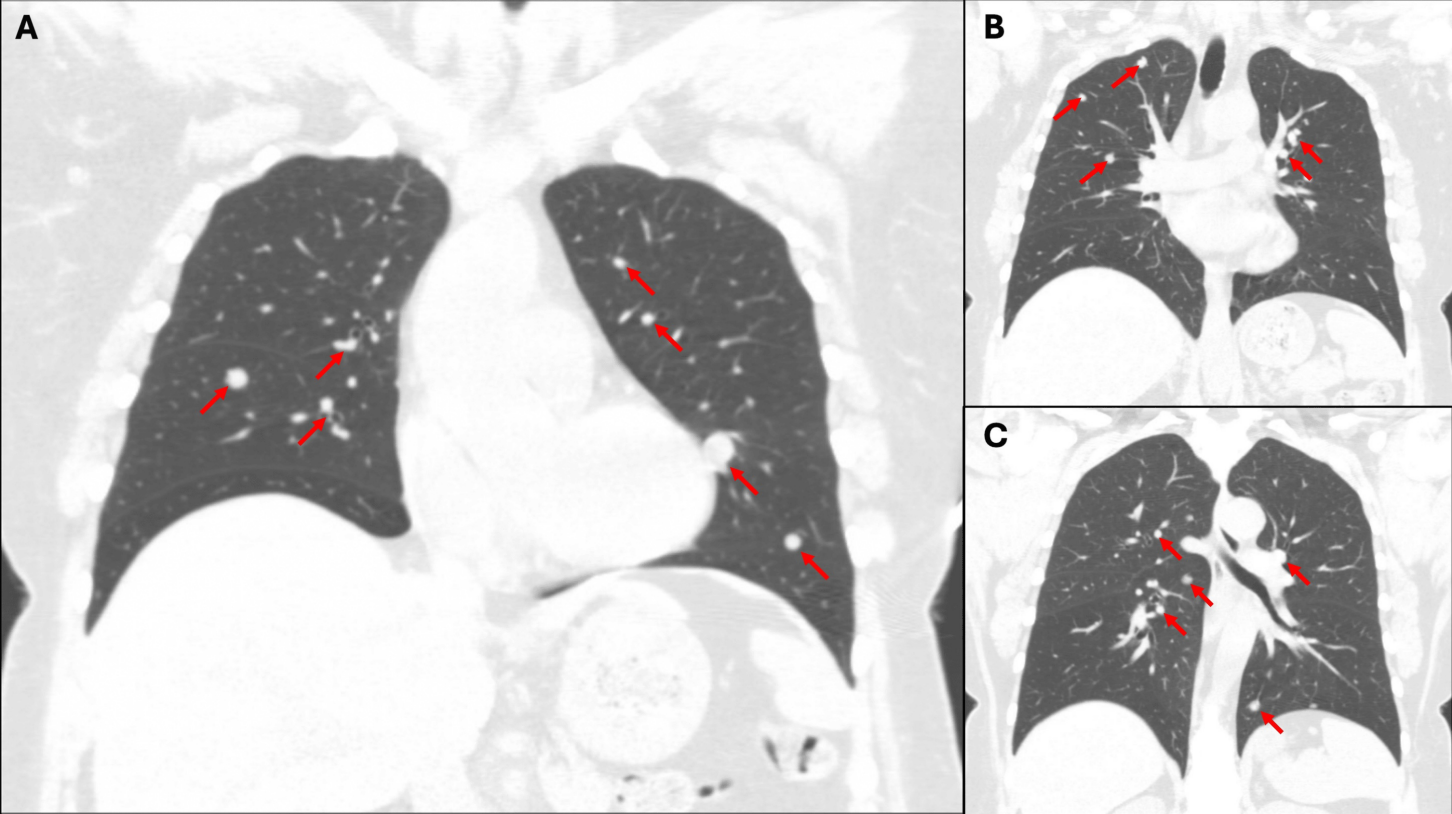
Computed tomography imaging demonstrating numerous bilateral pulmonary nodules. (a)-(c) Coronal reconstructions of the chest computed tomography. Representative pulmonary nodules are indicated using red arrows.

A 50-year-old female with a past medical history of hypertension and menorrhagia with no known prior history of uterine leiomyomas presented to her primary care physician for a nine-day history of worsening productive cough with yellow and green-tinged sputum along with congestion. A chest x-ray revealed several nodular densities throughout the bilateral lungs, greatest at the left lung base (Figure 1). Follow-up computed tomography to evaluate for possible malignancy with metastasis to the lungs redemonstrated diffuse bilateral pulmonary nodules, with the largest in the medial left lung measuring up to 2.8 cm, again concerning for pulmonary metastases (Figure 2). Further workup recommendations consisted of a positron emission tomography scan to evaluate for additional metastatic lesions as well as tissue sampling of a pulmonary lesion.

**Figure 3:**
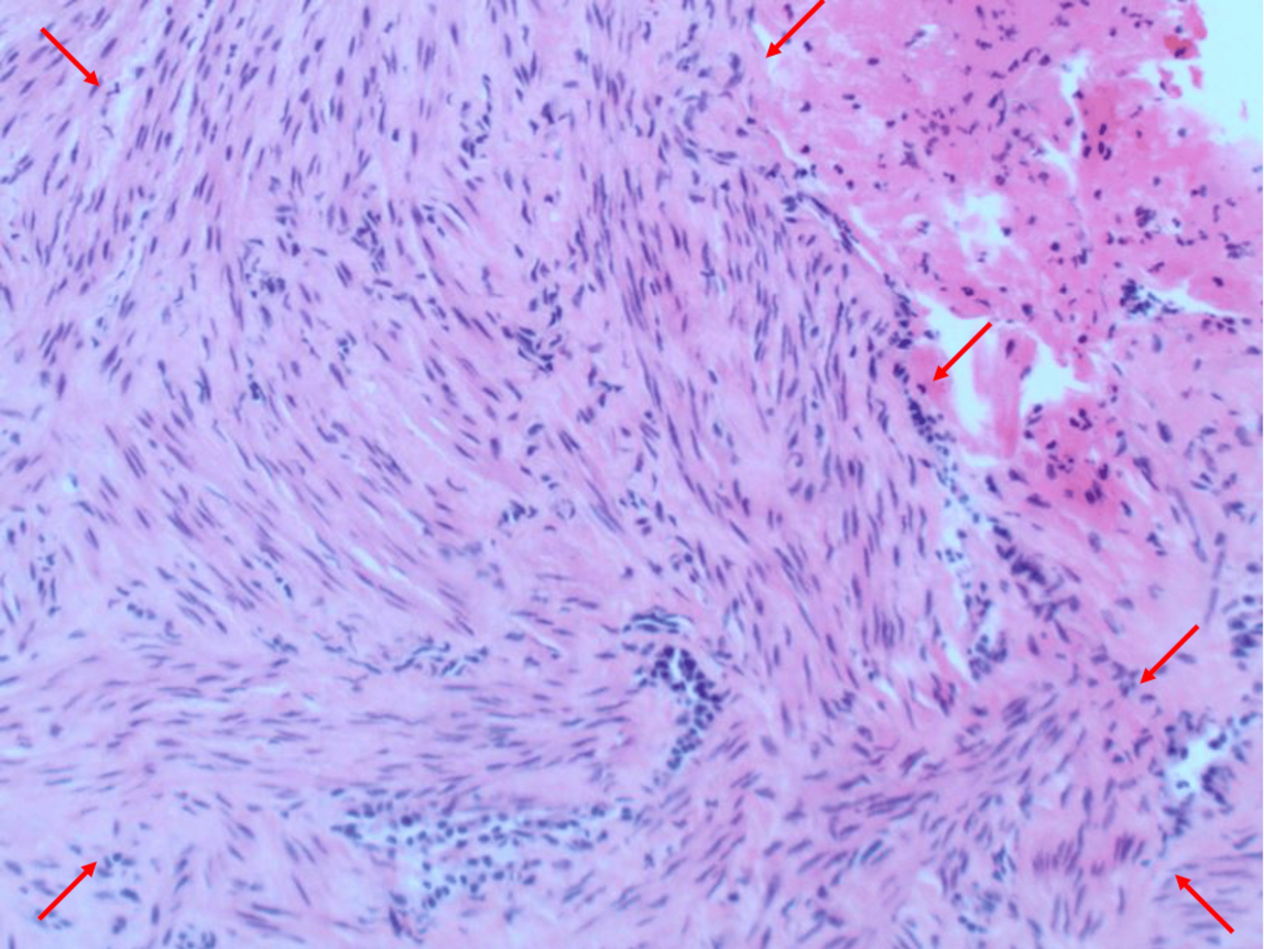
Pathological image of the right upper lung nodule obtained during the biopsy of the lesion. Image demonstrates intersecting fascicles of bland smooth muscle cells with abundant eosinophilic cytoplasm and haphazardly arranged. Hematoxylin and eosin stain was used, and the image was captured at 20x magnification. Smooth muscle fascicles are outlined using red arrows.

**Figure 4:**
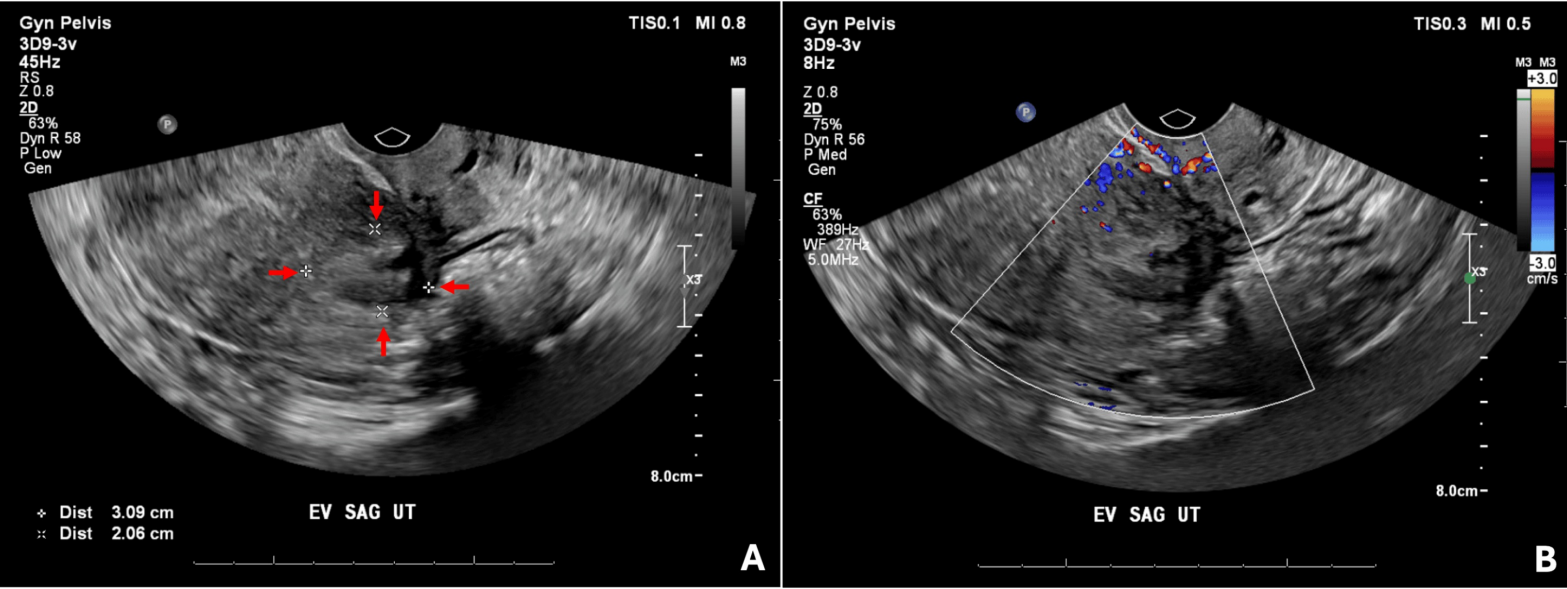
Transvaginal ultrasound imaging of the uterine leiomyoma. Images demonstrate the 3.09 cm x 2.06 cm x 2.08 cm intramural uterine leiomyoma in the lower uterine segment. (a) Fibroid outline from a sagittal image of the uterus indicated by the red arrows. (b) Fibroid on a sagittal image of the uterus with color Doppler.

**Figure 5:**
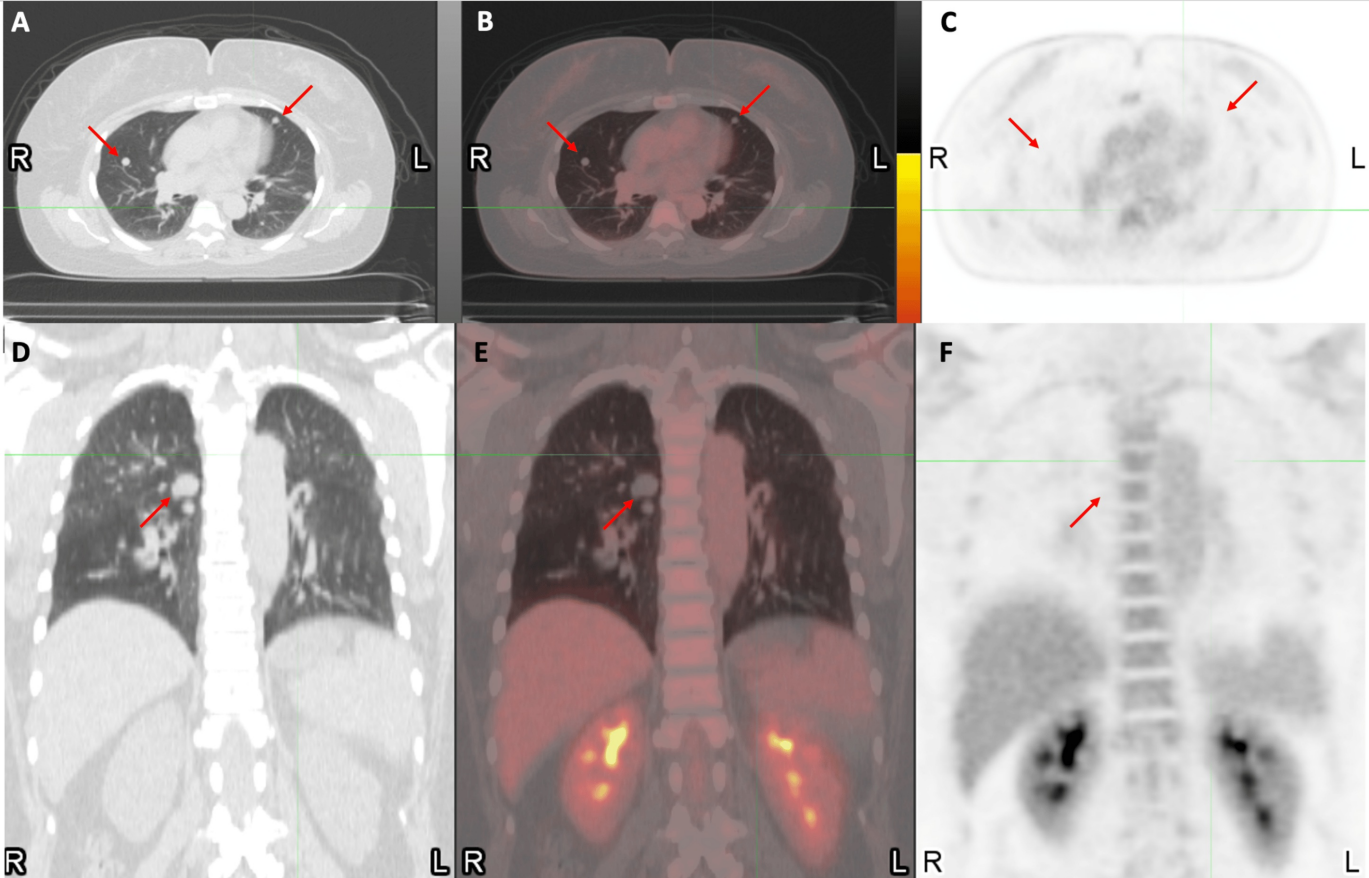
Positron emission tomography imaging of the thoracic cavity demonstrating the lack of hypermetabolism of the pulmonary nodules. Positron emission tomography imaging demonstrates no abnormal fluorodeoxyglucose uptake corresponding to the innumerable pulmonary nodules throughout the bilateral lungs. (a) Axial computed tomography of the thorax. (b) Axial fused positron emission tomography and computed tomography image of the thorax. (c) Axial attenuation corrected image of the thorax. (d) Coronal computed tomography of the thorax. (e) Coronal fused positron emission tomography and computed tomography image of the thorax. (f) Coronal attenuation corrected image of the thorax. Representative pulmonary nodule locations are indicated using red arrows.

The patient subsequently underwent bronchoscopy with transbronchial fine needle aspiration, transbronchial biopsy, and bronchoalveolar lavage of a right upper lung lesion. Pathology of the right upper lung lesion demonstrated smooth muscle fascicles that were non-mitotically active, haphazardly arranged, dotted with vascular slits, and lined with endothelial cells (Figure 3). Fine needle aspiration and bronchoalveolar lavage analysis resulted in no abnormal findings. The biopsy findings, along with the patient’s medical history significant for menorrhagia, favored the diagnosis of PBML. However, it was deemed impossible to definitively prove without a diagnosis of uterine leiomyoma. With PBML as the likely diagnosis, the patient underwent pelvic ultrasonography, which demonstrated a 3-cm intramural uterine fibroid in the lower uterine segment (Figure 4). Positron emission tomography scan did not demonstrate any hypermetabolism of the pulmonary nodules or any additional areas of abnormal hypermetabolism that would be concerning for metastatic disease (Figure 5). After several thoracic and gynecologic oncology tumor board discussions, the patient was referred to gynecologic oncology to discuss the surgical and medical treatment options for her likely diagnosis of PBML.

## Discussion

PBML is a rare asymptomatic condition with radiological features overlapping with other common serious medical conditions. PBML should be included in the differential diagnosis for reproductive-age females presenting with pulmonary nodules and a history of uterine leiomyoma. To avoid unnecessary imaging and procedures and to facilitate a fast accurate diagnosis, it is important for radiologists to understand the classic radiological findings of PBML on chest X-rays and computed tomography scans. Typical radiological features of PBML include well-circumscribed solitary or multiple pulmonary nodules that demonstrate homogeneous enhancement after intravenous contrast administration, ranging in size from a few millimeters to several centimeters, dispersed throughout the lungs and classically spare the pleura [2]. While these findings might also be expected with other etiologies, such as malignancy, sarcoidosis, and pulmonary granulomas, PBML is rarely associated with lymphadenopathy or calcifications [2]. Given these imaging characteristics, which significantly overlap with findings of other serious conditions, it is crucial to obtain a thorough medical history as well as order appropriate follow-up imaging to rule out malignant conditions before diagnosing PBML. This benign condition, despite its rarity, generally carries an excellent prognosis [2].

To date, there are no imaging characteristics that are specific for BML, and the diagnosis relies on histopathological examination of tissue samples from the uterus or the metastatic lesions. PBML lesions histologically demonstrate features of a benign neoplasm with a low index of tumor proliferation and a phenotype similar to smooth muscle [1]. During the workup of these suspicious lesions, it is crucial to rule out other spindle cell neoplasms, such as malignant melanoma, nerve sheath tumors, and sarcomas. The gynecological origin of the metastasized lesions can be confirmed by the abundance of estrogen and progesterone receptors [7].

PBMLs are commonly indolent, with some documented cases of spontaneous resolution [2]. Due to the rarity of BML, there is no consensus on the first-line treatment. A review of the literature describes treatment with surgical resection of the pulmonary lesions, oophorectomy, hormonal therapy, and chemical castration, with emphasis on tailoring treatment to each individual based on symptomatology and extent of disease [7]. 

Our case illustrates the importance of a multidisciplinary approach in promptly diagnosing PBML. As evidenced in the care of this patient, the interdisciplinary communication between various medical departments proved crucial in achieving a quick and accurate diagnosis for our patient. Specifically, family medicine was important in coordinating care of the patient, radiology was essential in identifying and reporting the lesions and offering recommendations for further workup based on the imaging findings, interventional pulmonology was utilized for bronchoscopy and tissue sampling, and pathology was required to evaluate the lesions histologically and ultimately provide the definitive diagnosis of BML. Finally, gynecologic oncology was recruited to treat the patient for PBML.

## Conclusions

This case highlights a rare medical condition and describes its classic radiological and histological findings that enable clinicians to achieve a prompt diagnosis for PBML, particularly useful for those who have known leiomyomas. While rare, PBML should be considered in the differential for women with multiple solid pulmonary lesions even without a known history of uterine fibroids, as presented in the case within this report. More importantly, this report highlights that while imaging and patient history might suggest PBML, histological sampling of the lesion is necessary to achieve a PBML diagnosis due to its non-specific radiological features. It is also important to note the significance of a multidisciplinary approach in managing patients with rare conditions such as the one described in this report. Finally, PBML is an uncommon disorder that poses a significant diagnostic challenge due to its rarity, general lack of symptomatology, and overlapping radiological findings similar to more common, yet serious, diseases. More research into PBML and its manifestations, clinically and radiologically, would be highly beneficial in educating physicians to achieve an early diagnosis and timely treatment without wastage of resources and excessive exposure to radiation.

## References

[1] Adair LB (2020). CT findings of pathology proven benign metastasizing leiomyoma. Radiol Case Rep.

[2] Abramson S, Gilkeson RC, Goldstein JD, Woodard PK, Eisenberg R, Abramson N (2001). Benign metastasizing leiomyoma: clinical, imaging, and pathologic correlation. AJR Am J Roentgenol.

[3] Barnaś E, Książek M, Raś R, Skręt A, Skręt-Magierło J, Dmoch-Gajzlerska E (2017). Benign metastasizing leiomyoma: a review of current literature in respect to the time and type of previous gynecological surgery. PLoS One.

[4] Taftaf R, Starnes S, Wang J, Shipley R, Namad T, Khaled R, Abdel Karim N (2014). Benign metastasizing leiomyoma: a rare type of lung metastases-two case reports and review of the literature. Case Rep Oncol Med.

[5] Del Real-Romo ZJ, Montero-Cantú C, Villegas-Cabello O (2014). Incidental benign metastasizing leiomyoma in a patient with bone sarcoma: a case report. Case Rep Surg.

[6] Ki EY, Hwang SJ, Lee KH, Park JS, Hur SY (2013). Benign metastasizing leiomyoma of the lung. World J Surg Oncol.

[7] Chouchane A, Boughizane S, Nouira M, Remadi S (2024). Benign metastasizing leiomyoma: new insights into a rare disease with an obscure etiopathogenesis. Diagn Pathol.

